# Risk of mental disorders and malnutrition in elderly COVID-19 survivors: An observational study

**DOI:** 10.12688/f1000research.121696.2

**Published:** 2023-10-02

**Authors:** Ria Maria Theresa, Marlina Dewiastuti, Sri Rahayu Ningsih, Lisa Safira

**Affiliations:** 1 Psychiatry, Universitas Pembangunan Nasional Veteran Jakarta, Jakarta, DKI, 12310, Indonesia; 2 Internal Medicine, Universitas Pembangunan Nasional Veteran Jakarta, Jakarta, DKI, 12310, Indonesia; 3 Biostatistik, Universitas Gunadarma, Depok, West Java, Indonesia; 4 Medical study program, Universitas Pembangunan Nasional Veteran Jakarta, Jakarta, DKI, 12310, Indonesia

**Keywords:** COVID-19, elderly, survivors, mental disorders, malnutrition

## Abstract

**Background:** The incidence rate of COVID-19 is around 11-15% in the elderly. The case fatality rate (CFR) of COVID-19 in the elderly is around 8.9% and increases with age. The risk of mental disorders and malnutrition is increased in COVID-19 survivors. Continuous inflammatory conditions result in a state of hypercatabolism that can disrupt brain neuroendocrine and protein consumption for the formation of acute-phase reactant proteins. Mental disorders and malnutrition can lead to fragility. The aim of this study was to assess the risk of mental disorders and malnutrition in elderly survivors of COVID-19.

**Methods:** This research was a cross-sectional study. The results of the research on age, disease symptoms, and comorbidities have proven that they are risk factors for mental disorders and malnutrition in elderly COVID-19 survivors. This study used total sampling and included 100 study subjects. The research was conducted in Depok for two months; data was collected directly through shared questionnaires and direct anthropometric measurements. The questionnaires used were the SRQ-20 tool for mental disorder screening and MNA for malnutrition screening.

**Results:** The risk factors for mental disorders were age over 70 years old OR 3 (CI 1.0-8.8), severe COVID-19 symptoms OR 4.5 (CI 1.2-16.17), and multi-comorbidity OR 2.3 (CI 0.6-8.8). The risk factors for malnutrition were age higher than 70 years old OR 2.5 (CI 0.8-7.9), moderate COVID-19 symptoms OR 6.3 (CI 2.0-19.81), and multi-comorbidity OR 6.6 (CI 1.5-28.5).

**Conclusions:** Those infected with COVID-19 have a risk of mental disorders and malnutrition, especially in geriatrics, and this risk increases with age.

## Introduction

The Coronavirus disease 2019 (COVID-19) is currently a worldwide pandemic. The morbidity and mortality rates are still increasing, especially in developing countries such as Indonesia. More than one million Indonesians suffer from COVID-19. Data from several studies indicate high morbidity and mortality rates in the elderly population. The incidence rate of COVID-19 in the population is around 11-17%, while the case fatality rate (CFR) is around 8.9% and increases with age. ^1^^–^^5^

The elderly population has an immunosenescence state, which causes disturbances in innate and adaptive immune systems. COVID-19 in the elderly results in a continuous inflammatory response, causing the clinical symptoms of COVID-19 in the elderly to often be more severe and the mortality rate to be high. ^6^

Survivors of COVID-19 have an increased risk of psychiatric disorders. About 20% of COVID-19 survivors will experience mental disorders. The most common clinical manifestations are depression, anxiety, and sleep disturbances. ^6^^–^^10^

Mental disorders increase along with the increasing age of COVID-19 survivors. The pathogenesis of psychiatric disorders in COVID-19 survivors is a continuous inflammatory state that can result in disturbances in neuroendocrine, neuroimmune, and nervous structures. ^11^^–^^15^

A total of 52.7% of the elderly population who were infected by COVID-19 suffers from malnutrition. This is due to the fact that most elderly people who suffer from COVID-19 have multiple comorbidities. In addition, the inflammatory reaction causes high catabolism, causing high protein consumption for the formation of acute phase reactants. The expression of angiotensin-2 receptor (ACE-2) in the gastrointestinal tract is high which causes symptoms of nausea, vomiting, diarrhea, and reduced appetite to increase in the elderly. The ongoing inflammatory reaction that occurs in the elderly results in a higher risk of malnutrition even when they have survived COVID-19. ^16^^–^^20^

Factors that influence mental disorders and malnutrition in COVID-19 survivors will be investigated in this study. Including gender, employment status, inpatient or outpatient care during illness, degree of illness, presence of caregiver, and vaccination status. There is no research was conducted on the factors that influence mental disorders and malnutrition in elderly COVID-19 survivors.

**Figure 1.**
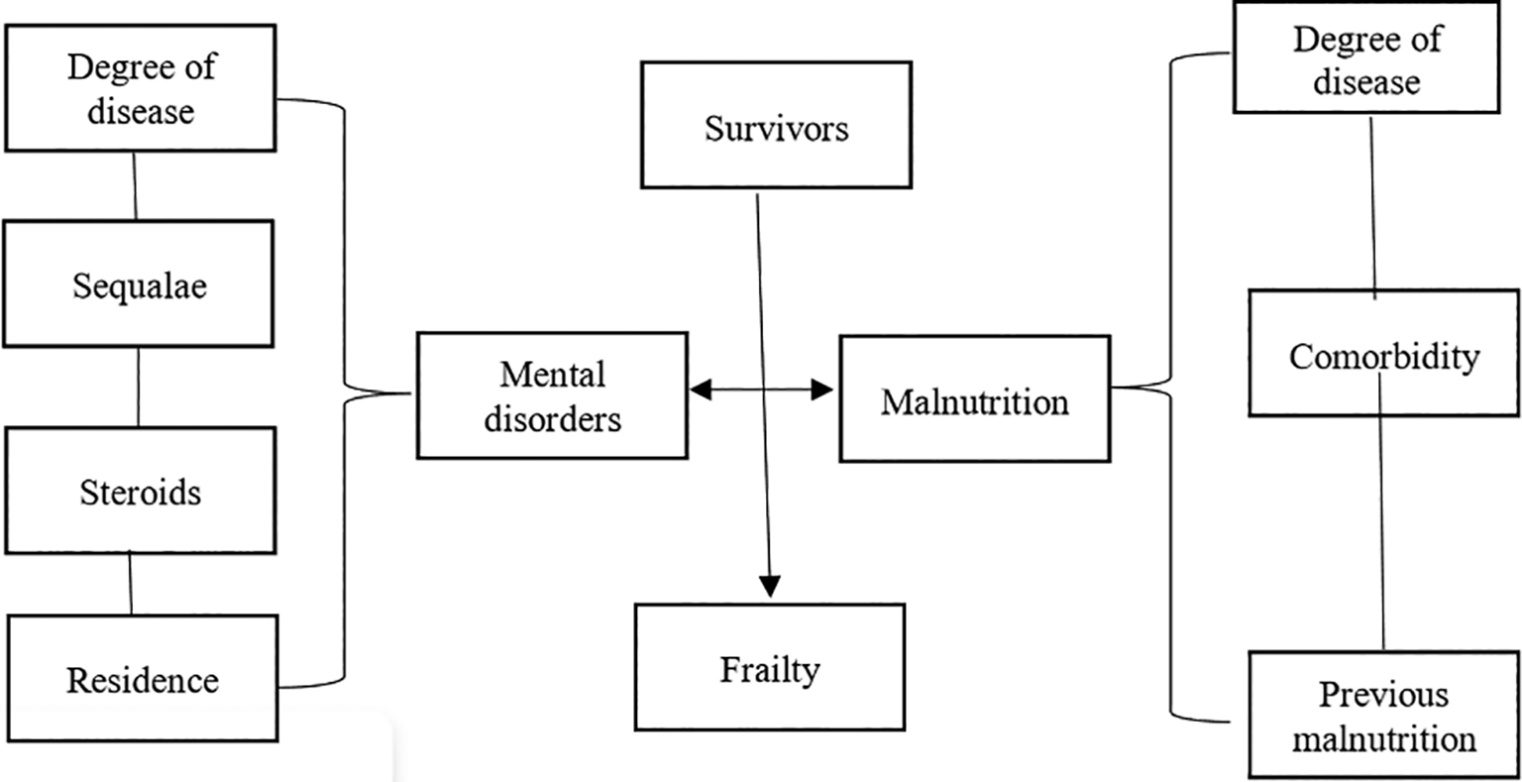
Risk of mental disorders and malnutrition in elderly COVID-19 survivors.

The risk of mental disorders and malnutrition in elderly COVID-19 survivors can lead to fragility. The fragility of elderly COVID-19 survivors increases hospitalization rates and mortality ( Figure 1). ^17^^,^^21^^–^^25^

The aim of this study was to investigate the risk of mental disorders and malnutrition in elderly COVID-19 survivors.

## Methods

### Ethical considerations

This study was approved by the UPN Veteran Jakarta Ethical Clearance Committee (Protocol number: 455/X/2021/KEPK) after due consultation, consent letters had been provided by the researchers to all respondents.

### Research methods

This research was a cross-sectional study conducted in Depok, Indonesia. Depok is an urban city that supporting the capital city. The elderly population is 3.77% of the total Depok population. The research sampled 100 people, patients diagnosed with COVID-19 by hospital data from Depok Hospital and willing to take part in the research (male = 52, female = 48) using total sampling. Data were collected by interview and direct data collection within three months of respondents being infected with COVID-19. The data was taken from the elderly population of Depok who had been infected with COVID-19 between May to July 2021. Inclusion criteria were the population of people over 60 years old and first time diagnosed with COVID-19 who were undergoing treatment in a hospital or quarantine at home, while exclusion criteria were the population who had been diagnosed with mental disorders and malnourished. Medical conditions such as dementia or other cognitive disorders were not previously evaluated in the study.

The outcome variables of this study were the risk of mental disorders and malnutrition. Both variables were categorized as having risks and having no risks. The independent variable in the study was age, measured since the respondent's birth. Another variable was the degree of severity of COVID-19; the severity of the disease was characterized as mild, moderate, and severe, and whether there were concomitant diseases or not. ^1^ The three variables were observed and tested using a logistic regression test. Insignificant variable expenditure causes a change in odds ratio (OR); if the OR change is more than 10% then the variable is a confounder variable and must be included in the model. Other potential confounders not observed in the study were economic status and a history of previous mental disorders.

Data were collected from questionnaires and anthropometric measurements. Questionnaire interviews were conducted by trained interviewers (RM). Interviews and data collection took 20-30 minutes per participant. Responses to questionnaires were inputted into electronic data. Before the data was collected, interrater reliability was carried out.

Sociodemographic information and health conditions obtained in this study included age, occupation, availability of caregivers, already received vaccinations, symptoms of COVID-19, and comorbidities. Data on comorbidities was obtained from self-reports.

Anthropometric measurements included measurements of height, weight, and body mass index. Measurement of body mass index was calculated based on the weight in kilograms divided by height in meters squared.

The data was cleaned after collection. Incomplete questionnaire data at the time of collection was re-confirmed with the study respondents.

The mental disorder questionnaire was based on the SRQ-20 questionnaire. There were 20 questions that were asked by direct interview. Obtaining a value of more than or equal to 8 meant the respondent had a risk of mental disorders. This questionnaire is a screening questionnaire that has been tested for validity and reliability and a diagnostic test with 88% sensitivity for mental disorder screening. ^26^^–^^28^

The malnutrition questionnaire used a mini nutritional assessment (MNA) questionnaire, conducted directly with interviews and direct body index measurements. Obtaining a value less than 11 was considered possible malnutrition. The MNA questionnaire is a questionnaire commonly used for malnutrition screening in the elderly. This questionnaire has been conducted with validity and reliability tests and diagnostic tests with a sensitivity of 96%. ^29^^,^^30^

Statistical analysis was performed with the Statistical Package for Social Sciences (SPSS). Significance was determined with an alpha value of <0.05. Descriptive analysis was conducted to look at demographic data such as gender, occupation, care during COVID-19, availability of caregivers, and vaccination status. Variables such as age, the severity of disease, and comorbidities were assessed using a Chi-square test. A logistic regression test was conducted to determine the factors that influence mental disorders and malnutrition with a multivariate model.

## Results

**Figure 2.**
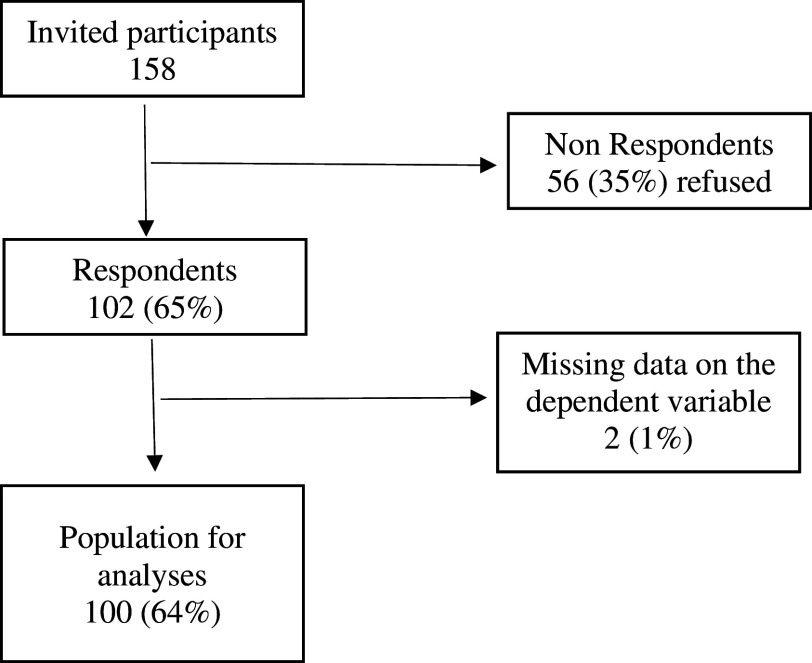
Participant inclusion chart.

This study invited 158 participants; 56 participants refused and did not respond the questionnaire. Two had missing data on the dependent variable and could not be contacted for confirmation of research data. A total of 100 participants were studied ( Figure 2).

A total of 100 respondents were tested based on baseline characteristics; p-values were over 0.05, so it can be concluded that there was no significant differences in the characteristics of respondents. The variable of availability of caregivers or nurses while being infected with COVID-19 for those who have a disorder did not show any significant differences; this is because the number of respondents who were outpatients is small.

As seen in Table 1, there was no difference in the occurrence of mental disorders between sexes; men and women had almost the same percentages. There was no difference in the occurrence of mental disorders between the different professions, nor was there a difference in the occurrence of mental disorders between those with caregivers and those without. Regarding treatment, a difference was observed because most of the elderly population infected with COVID-19 are likely to receive treatment in hospitals.

**Table 1.** Demographic description of respondents by mental disorder status.

Variable	Mental disorders		Total (%)	P-value
	Yes (%)	No (%)		
**Gender**				
Male	24 (46.20)	28 (53.80)	52 (100.00)	0.423
Female	26 (54.20)	22 (45.80)	48 (100.00)	
**Profession**				
Housewife	22 (55.00)	18 (45.00)	40 (100.00)	0.706
Retired	25 (46.30)	29 (53.70)	54 (100.00)	
Employee	3 (50.00)	3 (50.00)	6 (100.00)	
**Inpatient/outpatient**				
Outpatient	7 (26.90)	19 (73.10)	26 (100.00)	0.016
Inpatient	43 (58.10)	31 (41.90)	74 (100.00)	
**Caregiver**				
Yes	47 (49.00)	49 (51.00)	96 (100.00)	0.307
No	3 (75.00)	1 (25.00)	4 (100.00)	
**Income**				
<Rp. 5.000.000	35 (50.00)	35 (50.00)	70 (100.00)	0.999
**≥** Rp. 5.000.000	15 (50.00)	15 (50.00)	30 (100.00)	
**Vaccinated status**				
Done	10 (38.50)	16 (61.50)	26 (100.00)	0.171
None	40 (54.10)	34 (45.90)	74 (100.00)	

The characteristics of the malnourished group also showed there was no difference in baseline characteristics (gender, profession, caregiver, income, vaccinated status), where the characteristics of the respondents in patients who had malnutrition and those who did not suffer from malnutrition were homogeneous.

In Table 2 there are no differences between gender, profession, and availability of caregivers’ effect on the risk of malnutrition. However, there was a difference between treatment during COVID-19 infection and the risk of malnutrition; this may be because most elderly people infected with COVID-19 get treatment in hospitals.

**Table 2.** Demographic description of respondents based on malnutrition disorder status.

Variable	Malnutrition		Total (%)	P-value
	Yes (%)	No (%)		
**Gender**				
Male	25 (48.10)	27 (51.90)	52 (100.00)	0.543
Female	26 (54.20)	22 (45.80)	48 (100.00)	
**Profession**				
Housewife	21 (52.50)	19 (47.50)	40 (100.00)	0.970
Retired	27 (50.00)	27 (50.00)	54 (100.00)	
Employee	3 (50.00)	3 (50.00)	6 (100.00)	
**Inpatient/outpatient**				
Outpatient	6 (23.10)	20 (76.90)	26 (100.00)	0.001
Inpatient	45 (60.80)	29 (39.20)	74 (100.00)	
**Caregiver**				
Yes	47 (49.00)	49 (51.00)	96 (100.00)	0.136
No	4 (100.00)	0 (00.00)	4 (100.00)	
**Income**				
<Rp. 5.000.000	35 (50.00)	35 (50.00)	70 (100.00)	0.760
**≥** Rp. 5.000.000	16 (53.30)	14 (46.70)	30 (100.00)	
**Vaccinated status**				
Done	11 (42.30)	15 (57.70)	26 (100.00)	0.171
None	40 (54.10)	34 (45.90)	74 (100.00)	

Based on the results in Tables 3 and 4, the variables of age, symptoms, and comorbidities were included in the multivariate analysis, both for mental disorders and malnutrition disorders.

**Table 3.** Relationship variables age, symptoms, and comorbidities with mental disorders.

Variable	Mental disorders		Total (%)	P-Value
	Yes (%)	No (%)		
**Age**				
60-70 yo	30 (41.10)	43 (58.90)	73 (100.00)	0.003
>70 yo	20 (74.10)	7 (25.90)	27 (100.00)	
**Symptoms**				
Mild	12 (30.80)	27 (69.20)	39 (100.00)	0.005
Moderate	25 (58.10)	18 (41.90)	43 (100.00)	
Severe	13 (72.20)	5 (27.80)	18 (100.00)	
**Comorbidities**				
None	15 (36.60)	26 (63.40)	41 (100.00)	0.042
1 Comorbidity	24 (58.50)	17 (41.50)	41 (100.00)	
>1 Comorbidity	11 (61.10)	7 (38.90)	18 (100.00)	

**Table 4.** Relationships between variables of age, symptoms, and comorbidities with malnutrition disorder.

Variable	Malnutrition		Total (%)	P-value
	Yes (%)	No (%)		
**Age**				
60-70 yo	31 (42.50)	42 (57.50)	73 (100.00)	0.005
>70 yo	20 (74.10)	7 (25.90)	27 (100.00)	
**Symptoms**				
Mild	11 (28.20)	28 (71.80)	39 (100.00)	0.001
Moderate	27 (62.80)	16 (37.20)	43 (100.00)	
Severe	13 (72.20)	5 (27.80)	18 (100.00)	
**Comorbidities**				
None	12 (29.30)	29 (70.70)	41 (100.00)	0.001
1 Comorbidity	27 (65.90)	14 (34.10)	41 (100.00)	
>1 Comorbidity	12 (66.70)	6 (33.30)	18 (100.00)	

Table 3 shows there was a relationship between increased age and the risk of mental disorders. There was also a relationship between the severity of COVID-19 symptoms and the number of comorbidities with the risk of mental disorders.

Table 4 shows a relationship between increasing age and the risk of malnutrition. There was also a relationship between the severity of COVID-19 symptoms and the number of comorbidities with the risk. Comorbidities that affect degree of disease were hypertension, diabetes mellitus, and cardiovascular disease (coronary arterial disease or cerebrovascular disease.

Based on the results of the multiple logistic regression analysis in Table 5, the factors that influenced mental disorders were age and symptoms. People older than 70 years had a three-time greater risk of experiencing mental disorders than the elderly aged between 60-70 years old, after controlling for symptoms and comorbidities variables at a 95% confidence interval (CI) between 1,071 to 8,83. The elderly with severe COVID-19 symptoms were at a 4.5-time greater risk of experiencing mental disorders compared to the elderly with mild symptoms, after controlling for age and comorbidities variables at a 95% confidence level between 1.23 to 16.71.

**Table 5.** Mental disorder multiple logistic regression model.

Variable	P-value	OR	95% Confidence interval	
			Lower	Upper
**Age**				
60-70 yo*				
>70 yo	0.037	3.085	1.071	8.883
**Symptoms**				
Mild*	0.022			
Moderate	0.021	3.219	1.195	8.671
Severe	0.023	4.540	1.23	16.71
**Comorbidities**				
None*	0.335			
1 Comorbidity	0.223	1.872	0.684	5.124
>1 Comorbidity	0.202	2.359	0.631	8.812

Key: * reference group.

Based on the results of multivariate analysis in Table 5, malnutrition disorders were influenced by symptom variables and comorbidities. Elderly people with more than one comorbidity had a 6.6-time greater risk of experiencing malnutrition after controlling for symptoms and age variables at a 95% confidence level, between 1.56 to 28.57.

## Discussion

One-third of the post-COVID-19 population experience mental disorders; 40% of patients will experience depression, and the rest will experience symptoms such as anxiety, and delirium. ^10^^,^^11^^,^^23^^,^^31^^-^^33^ Between 43-70% of COVID-19 survivors experience psychological disorders. Several studies say this is related to the degree of disease, age, and comorbidities. However, several studies have stated that mental disorders are not related to this, especially in the elderly. ^5^^,^^6^^,^^14^^,^^15^

The older the age, the higher the risk for mental disorders will be. Based on the results of our study, it was found that as age increased, the risk of mental disorders in the elderly after COVID-19 infection increased by 2.5 times according to the results of the study in Tables 3 and 5. ^16^

COVID-19 infection predisposes to mental disorders, which are induced by cytokine bodies and hyperinflammatory states. ^10^^,^^11^ Therefore, it can cause disruption of the blood-brain barrier and ultimately inflammation of the nervous system. In the elderly, there is a susceptibility to inflammation. ^17^ Hyper-inflammatory conditions affect the severity of COVID-19 disease; the severity of COVID-19 disease will increase the risk of post-infection mental disorders. In addition, comorbidity in the elderly is often multi-comorbid. ^17^ The number of comorbidities increases the risk of mental disorders. ^23^^,^^32^^,^^34^ Table 3 shows that there is a relationship between age, the degree of disease severity, and comorbidities that increase the risk of mental disorders in the elderly after COVID-19 infection. The degree of severe illness leads to a 4.5 times higher risk of mental disorders and multi comorbidities lead to a 2.3 times higher risk of experiencing mental disorders. ^34^ Interferon-gamma related to hyperinflammatory condition increase during covid-19 infection can affect brain function. it is well-known that chronic accumulation of cytokines causes neuronal damage. ^2^^,^^3^

Malnutrition is a nutritional disorder that has an unfavorable impact, especially on the elderly. The incidence of malnutrition in the elderly infected with COVID-19 is higher than in the general population. The pathomechanism of malnutrition is an acute inflammatory state causing high body protein consumption, and less lean body mass in the elderly, which continues to decrease with increasing age so that elderly people often lose weight due to acute inflammation. The infection of SARS-CoV-2 in the gastrointestinal system of the elderly is greater, so elderly people who are infected with COVID-19 often experience severe gastrointestinal disorders. Other factors can also influence malnutrition: the severity of COVID-19 infection increases the risk of malnutrition as 32.3% of the elderly infected with COVID-19 will continue to be malnourished 30 days after infection. Comorbidity in the elderly is also related to the incidence of malnutrition, which is related to chronic inflammation that leads to acute exacerbations causing a hyperinflammatory state so that catabolism increases and muscle mass is used. ^10^^,^^12^^,^^18^^,^^19^^,^^35^^,^^36^

Based on the results of the study, it was found that older age, degree of disease severity, and comorbidities were associated with the risk of malnutrition in the elderly after COVID-19. Increasing age increased the risk of malnutrition by 2.5 times. The severity of the disease also increased the risk of malnutrition, although in this study it was shown that people with the moderate disease had the highest risk of malnutrition, which was 6.3 times higher, while people with severe degree disease had 4.4 times risk of malnutrition based on data number of participants with moderate symptoms is more than severe symptoms and patients with severe symptoms had died during hospitalization. In some studies, 32.3% of patients suffering from malnutrition during treatment still experienced malnutrition on day 30, meaning about 70% experienced an improvement in their condition. In this study, multi-comorbidities led to 6.6 times higher risk of malnutrition, more comorbidities, and increased susceptibility in the elderly as seen in Table 6. ^10^^,^^19^^–^^22^

**Table 6.** Malnutrition disorder multiple logistic regression model.

Variable	P-Value	OR	95% Confidence interval	
			Lower	Upper
**Age**				
60-70 yo*				
>70 yo	0.114	2.515	0.800	7.906
**Symptoms**				
Mild*	0.003			
Moderate	0.001	6.368	2.047	19.812
Severe	0.029	4.420	1.163	16.802
**Comorbidities**				
None*	0.007			
1 Comorbidity	0.005	5.045	1.645	15.472
>1 Comorbidity	0.010	6.685	1.56	28.57

Key: * reference group.

## Conclusions

Age, COVID-19 symptoms and the presence of disease comorbidities are risk factors for mental disorders and malnutrition in COVID-19 elderly survivors. The older the age, the more severe the symptoms of COVID-19; the number of comorbidities also increased the risk of mental disorders and malnutrition.

Evaluation of mental health and nutritional status in elderly COVID-19 survivors needs to be carried out regularly to avoid vulnerabilities which will negatively impact the quality of life of elderly people.

The limitations of this study are the total sampling approach. In addition, in this study some variable confounders couldn’t be strictly controlled. Variable confounders that can affect the results include mental disorders that have previously been experienced or have had previous symptoms.

## Data availability

### Underlying data

Figshare: Risk of mental disorders and malnutrition in elderly COVID-19 survivors, https://doi.org/10.6084/m9.figshare.19588519.v2. ^37^

This project contains the following underlying data:

- response from 100 respondents in Covid-19 Survivor.csv

Data are available under the terms of the Creative Commons Attribution 4.0 International license (CC-BY 4.0).
