# Supplementary material for: Risk of mental disorders and malnutrition in elderly COVID-19 survivors: An observational study
Source: F1000Res. 2023 Oct 2;12:42. Originally published 2023 Jan 11. [Version 2] doi: 10.12688/f1000research.121696.2 (PMC12853014; doi:10.12688/f1000research.121696.2)
Supplement: Peer review history

# Reviewer report: Reviewer response for version 2

**DOI:** 10.5256/f1000research.155800.r449692

**Published:** 2026-01-28

**Authors:** Yuni Asri

**Affiliations:** 1 Institut Teknologi Sains dan Kesehatan RS dr. Soepraoen Malang, Malang, Malang, Indonesia

**1. Clarity of presentation and use of current literature**

 Assessment: Partly

 The manuscript is generally well structured, and the results are presented in a logical sequence. Tables and figures are readable, and the analytical approach is transparent at a basic level. However, there are notable concerns:

1. The authors repeatedly claim that *“there is no research”* examining factors associated with mental disorders and malnutrition in elderly COVID-19 survivors. This statement is factually inaccurate. Multiple studies and systematic reviews published between 2021–2023 have already addressed mental health outcomes and malnutrition in older COVID-19 populations.
2. Although some recent references are cited, the manuscript does not adequately contextualize the study within the existing body of evidence, nor does it clearly articulate what is novel about the present work (e.g., regional context, combined outcomes, or post-acute timeframe).

To ensure the scientific integrity of the work, it is essential to revise the introduction so that it accurately represents the current body of literature, steering clear of making absolute statements. It is also important to clearly identify the specific research gap, such as the joint evaluation of mental and nutritional risks among elderly Indonesian survivors within three months after infection. Additionally, the discussion should be enhanced by directly comparing the results with recent studies conducted both internationally and regionally.

**2. Study design and technical soundness**

 Assessment: Partly

 A cross-sectional design is acceptable for exploring associations between risk factors and outcomes. The inclusion and exclusion criteria are stated, and the use of validated screening tools is appropriate for a preliminary risk assessment.

 While the study's design does not allow for causal conclusions, causal language is frequently used in the discussion and conclusions. There is a possibility of selection bias because participation was based on individuals' willingness to respond, and those experiencing mental health or nutritional issues might have been more likely to participate. Furthermore, important potential confounding factors, such as socioeconomic status, existing cognitive impairments, and prior subclinical mental health symptoms, were identified but not thoroughly examined in the analysis.

 Key issues to address include: reframing interpretations solely as associations rather than causal links, explicitly acknowledging selection bias and residual confounding as significant limitations, and ensuring that conclusions accurately reflect the limitations inherent in a cross-sectional design.

**3. Sufficiency of methodological detail for replication**

 Assessment: Partly

 The manuscript describes data collection procedures, instruments used, and general analytical methods. Nonetheless, several elements necessary for full replication are insufficiently detailed:

1. The rationale for dichotomizing age at 70 years is not justified clinically or statistically.
2. The variable selection strategy for multivariate logistic regression (e.g., criteria for inclusion, handling of collinearity) is not fully explained.
3. It is unclear how missing data (if any beyond the two excluded participants) were handled.

Key issues to address include offering a clear rationale for the chosen age categorization or exploring alternative approaches such as continuous age modeling or sensitivity analysis. Additionally, it is important to elaborate on the regression modeling strategy and clarify data management procedures to improve reproducibility.

**4. Statistical analysis and interpretation**

 Assessment: Partly

 The choice of chi-square tests and logistic regression for binary outcomes is appropriate. Reporting of odds ratios with 95% confidence intervals is adequate.

 Nevertheless: Some confidence intervals are broad, suggesting a lack of statistical power, which should be more clearly acknowledged. Multicomorbidity is considered as a count variable, yet the specific impact of individual comorbidities (such as hypertension, diabetes, and cardiovascular disease) is not examined. The discussion sometimes exaggerates the interpretation of odds ratios in relation to the sample size and the precision of the estimates.

 Key considerations include moderating the interpretation of effect sizes, particularly when confidence intervals are broad, and discussing the consequences of limited statistical power. If possible, conduct an exploratory analysis of individual comorbidities or provide a clear rationale for not doing so.

**5. Availability of source data**

 Assessment: Yes

 The authors have shared the foundational dataset on Figshare, promoting transparency and reproducibility in accordance with F1000Research guidelines. This aspect enhances the study's credibility.

**6. Support of conclusions by results**

 Assessment: Partly

 The conclusions are generally aligned with the reported results. However, they are framed too strongly given the study design and limitations.

1. Statements implying increased “risk” in a causal sense should be revised to “association.”
2. Recommendations for clinical practice, while reasonable, should be presented more cautiously and framed as suggestions for screening and further research rather than definitive guidance.

Key issues to address: Revise conclusions to accurately represent associative results. Ensure that recommendations align with the strength of the evidence.

Is the work clearly and accurately presented and does it cite the current literature?

Partly

If applicable, is the statistical analysis and its interpretation appropriate?

Partly

Are all the source data underlying the results available to ensure full reproducibility?

Yes

Is the study design appropriate and is the work technically sound?

Partly

Are the conclusions drawn adequately supported by the results?

Partly

Are sufficient details of methods and analysis provided to allow replication by others?

Partly

Reviewer Expertise:

My research areas include Public Health and Community Health Nursing, with a particular focus on gerontology, mental health, elderly care, and health services research. My work explores population health outcomes, determinants of health among older adults, mental well-being in vulnerable groups, and the development and evaluation of nursing interventions in both community and clinical settings.

I confirm that I have read this submission and believe that I have an appropriate level of expertise to confirm that it is of an acceptable scientific standard, however I have significant reservations, as outlined above.

# Reviewer report: Reviewer response for version 2

**DOI:** 10.5256/f1000research.155800.r279772

**Published:** 2024-06-10

**Authors:** Yan-Jiang Wang, Yu-Hui Liu

**Affiliations:** 1 Department of Neurology and Centre for Clinical Neuroscience, Daping Hospital, Third Military Medical University, Chongqing, China; 2 Department of Neurology and Clinical Center for Neuroscience, Daping Hospital, Third Military Medical University, Chongqing, Chongqing, China

This study aimed to examine the risk of mental disorders and malnutrition among older individuals who survived COVID-19. The findings revealed that older age, severe COVID-19 infection, and the presence of multiple comorbidities were linked to adverse mental health outcomes and malnutrition following the infection. The study was conducted in a meticulous manner; however, a few concerns need to be addressed.

**-Introduction**

1. The authors should provide a clear explanation for why they chose to investigate both mental health disorders and malnutrition in a single study, as these two outcomes may not appear to be directly related.
2. The authors claimed that “There is no research was conducted on the factors that influence mental disorders and malnutrition in elderly COVID-19 survivors”. This should be approached with caution because there is a significant amount of research on this topic.

Tosato M, et.al., 2023 (Ref 1)

 Griffiths J et.al., 2023 (Ref 2) 

 Boaz M. et.al., 2023 (Ref 3)

 Feng X et.al., 2022 (Ref 4) 

**- Methods**

1. It is important to acknowledge the potential bias in participant recruitment. Individuals who have experienced mental health complaints and malnutrition may be more inclined to participate, whereas those in good health may have less interest in participation.
2. Age was considered as a dichotomous variable in the regression models. However, it is important for the authors to provide an explanation for their selection of 70 years as the threshold to differentiate between the older and younger age groups.
3. Using the SRQ-20 questionnaire for assessment was too simplistic and did not provide a clear indication of the specific type of mental health disorders among older COVID-19 survivors. This limitation should be acknowledged.

**- Results**

 The authors observed that multicomorbidities were linked with both mental health disorders and malnutrition. However, it's crucial to identify the primary contributing comorbidity to these health outcomes. I would recommend the authors to analyze the association between each comorbidity and the outcomes separately

**- Discussion**

1. In a cross-sectional investigation, establishing causative associations between age, disease severity, comorbidities, and the health consequences of COVID-19 is challenging. This limitation should be acknowledged, and further longitudinal investigations are necessary to address this gap.
2. The small sample size should be acknowledged as a limitation of the study.

Is the work clearly and accurately presented and does it cite the current literature?

Partly

If applicable, is the statistical analysis and its interpretation appropriate?

Partly

Are all the source data underlying the results available to ensure full reproducibility?

Partly

Is the study design appropriate and is the work technically sound?

Partly

Are the conclusions drawn adequately supported by the results?

Partly

Are sufficient details of methods and analysis provided to allow replication by others?

Partly

Reviewer Expertise:

Neurology; Dementia; Covid-19

We confirm that we have read this submission and believe that we have an appropriate level of expertise to state that we do not consider it to be of an acceptable scientific standard, for reasons outlined above.

## References

[1] : Malnutrition in COVID-19 survivors: prevalence and risk factors. *Aging Clin Exp Res* .2023;35(10) : 10.1007/s40520-023-02526-4 2257-2265 10.1007/s40520-023-02526-4 37665556 PMC10520154

[2] : Malnutrition, Depression, Poor Sleep Quality, and Difficulty Falling Asleep at Night Are Associated with a Higher Risk of Cognitive Frailty in Older Adults during the COVID-19 Restrictions. *Nutrients* .2023;15(13) : 10.3390/nu15132849 10.3390/nu15132849 37447178 PMC10343894

[3] : Systematic Review and Meta-Analysis: Malnutrition and In-Hospital Death in Adults Hospitalized with COVID-19. *Nutrients* .2023;15(5) : 10.3390/nu15051298 10.3390/nu15051298 36904295 PMC10005527

[4] : Risk of Malnutrition in Hospitalized COVID-19 Patients: A Systematic Review and Meta-Analysis. *Nutrients* .2022;14(24) : 10.3390/nu14245267 10.3390/nu14245267 36558436 PMC9780808

# Reviewer report: Reviewer response for version 2

**DOI:** 10.5256/f1000research.155800.r211293

**Published:** 2023-10-17

**Authors:** Mirasari Putri

**Affiliations:** 1 Department of Biochemistry, Nutrition, and Biomolecular, Faculty of Medicine, Universitas Islam Bandung, Bandung, Indonesia

I think revisions are appropriate.

Is the work clearly and accurately presented and does it cite the current literature?

Yes

If applicable, is the statistical analysis and its interpretation appropriate?

Yes

Are all the source data underlying the results available to ensure full reproducibility?

Yes

Is the study design appropriate and is the work technically sound?

Yes

Are the conclusions drawn adequately supported by the results?

Yes

Are sufficient details of methods and analysis provided to allow replication by others?

Yes

Reviewer Expertise:

NA

I confirm that I have read this submission and believe that I have an appropriate level of expertise to confirm that it is of an acceptable scientific standard.

# Reviewer report: Reviewer response for version 1

**DOI:** 10.5256/f1000research.133591.r186579

**Published:** 2023-07-31

**Authors:** Tusawar Iftikhar Ahmad, Amina Alamgir

**Affiliations:** 1 The Islamia University of Bahawalpur Pakistan, Bahawalpur, Punjab, Pakistan; 2 Department of Economics, The Islamia University of Bahawalpur Pakistan, Bahawalpur, Punjab, Pakistan

Feedback for improving the quality of the manuscript:

1. In the abstract, include statistics on the incidence and case fatality rate (CFR) of COVID-19 in the elderly within the context of Indonesia.
2. In the abstract, present the findings related to Odds Ratios in the following format: for example, provide the numeric value of the OR (p-value = 0.00; CI = 1.0-8.8).
3. In Figure 1, the visualization of the sequence depicting the risk of mental disorders and malnutrition in elderly COVID-19 survivors is unclear. Please clarify the directions of the arrows and sequence of the text boxes to provide a more coherent understanding.
4. Please provide a statistical justification for the adequacy of the sample size (n = 100) and its implications for the generalizability of the findings.
5. The current study lacks a clear presentation of its theoretical underpinnings. Please include a section to address this aspect.
6. The research gap is not specifically identified. Consider highlighting the specific gap that this study aims to address.
7. Provide a brief description of the study area (Depok, Indonesia), including cartographic presentation, demography, socioeconomic dynamics, etc.
8. The paper mentions the use of the SRQ-20 and the MNA questionnaires to assess mental disorder and malnutrition, respectively. Please add a brief description of the structures of both questionnaires in the paper.
9. Please specify the sampling technique employed to collect the data.
10. Include a description, along with a mathematical expression, explaining the relevance of using multiple logistic regression as the estimation technique for this research.

Is the work clearly and accurately presented and does it cite the current literature?

Yes

If applicable, is the statistical analysis and its interpretation appropriate?

Yes

Are all the source data underlying the results available to ensure full reproducibility?

Yes

Is the study design appropriate and is the work technically sound?

Yes

Are the conclusions drawn adequately supported by the results?

Yes

Are sufficient details of methods and analysis provided to allow replication by others?

Partly

Reviewer Expertise:

Health Economics

We confirm that we have read this submission and believe that we have an appropriate level of expertise to confirm that it is of an acceptable scientific standard, however we have significant reservations, as outlined above.

# Reviewer report: Reviewer response for version 1

**DOI:** 10.5256/f1000research.133591.r164467

**Published:** 2023-05-02

**Authors:** Mirasari Putri

**Affiliations:** 1 Department of Biochemistry, Nutrition, and Biomolecular, Faculty of Medicine, Universitas Islam Bandung, Bandung, Indonesia

**Abstract**

1. Method: Write the time of this research conducted
2. Write down the significant comorbidities that play a role in mental disorders and malnutrition based on the findings in this study.

**Introduction:**

1. It should provide evidence gaps to highlight unanswered questions in this study and how this study will contribute to filling the gap.
2. Highlight the originality and novelty of this study.

**Method:**

1. Was it true that there were 100 elderly people affected by Covid in May-July 2021 throughout Depok? Explain further what is meant by the total sample in this study.
2. Why was malnutrition in the elderly before being diagnosed with Covid not excluded in this study? Malnutrition is one risk factor in the elderly with or without Covid. How can authors ensure that the patients' malnutrition is because of Covid, not because they have had it before?
3. Paragraph 2, line 2 adds a reference for mental disorder categorization.
4. Paragraph 2, line 4 adds the reference for Covid severity.
5. Write the number of ethical approval.
6. Paragraph 1, line 2 add the reference for mental disorder categorized and explain the method briefly
7. Explain whether the patients who are used as subjects were patients who experienced Covid for the first time, have been exposed to it more than once, or were all taken as subjects.

**Result:**

1. Explain further about the comorbidities presented in tables 3 and 4. In more than 1 comorbidity, which comorbidities are the high risk factors for mental disorder and malnutrition in your research?
2. Paragraph 10: which table represents multivariate analysis? Please write it.
3. In the table note, write the statistical method used and the total number of respondents.

**Discussion: **

1. Paragraph 3: Explain in more detail the mechanism of hyperinflammation and cytokines in infections associated with the nervous system, which are at risk of causing mental disorders
2. Paragraph  3: Explain in more detail about the multiple comorbidities that can increase the risk of mental disorders concerning the comorbidities found in your research and also the mechanism
3. Paragraph  4: the authors explained that gastrointestinal factors are one of the causes of malnutrition in the elderly with Covid. Did the authors investigate these gastrointestinal factors? If yes, the results can be written in the results section. Because this is something that can confirm the mechanism of malnutrition in the elderly with Covid
4. Paragraph 5: Discuss why the risk of malnutrition in Covid patients with moderate symptoms was higher than those with severe ones.

Is the work clearly and accurately presented and does it cite the current literature?

Yes

If applicable, is the statistical analysis and its interpretation appropriate?

Yes

Are all the source data underlying the results available to ensure full reproducibility?

Yes

Is the study design appropriate and is the work technically sound?

Yes

Are the conclusions drawn adequately supported by the results?

Yes

Are sufficient details of methods and analysis provided to allow replication by others?

Yes

Reviewer Expertise:

Nutrition-Immunology-Biomolecular

I confirm that I have read this submission and believe that I have an appropriate level of expertise to confirm that it is of an acceptable scientific standard, however I have significant reservations, as outlined above.

# Response

**Published:** 2023-06-21

**Authors:** Ria Maria Theresa

**Affiliations:** UPN Veteran Jakarta, Indonesia

Abstract : There is no cachexia and no cancer 

 Introduction:

 1. Malnutrition in elderly will worsen health status in any situation.

 2. No studies have assesed malnutrition in elderly covid patients.

 Method:

 1. Pasien was diagnosed covid-19 by hospital data from Depok hospital, using total sampling of hospital data.

 2. The malnourished patient was excluded in this study.

 We are sure that from this study, the sample was not diagnosed malnourished based on hospital data (BMI and by mini nutritional assessment from hospital data when patients were hospitalized).

 3.Paragraph 2, line 2 adds a reference for mental disorder categorization:

 Stein AMB: Coronavirus disease 2019 (COVID-19): Psychiatric illness. *Wolters Kluwer.* 2020; **2019**: 1–31.

 4.Paragraph 2, line 4 adds the reference for Covid severity:

 Azwar MK, Setiati S, Rizka A, *et al.*: Clinical Profile of Elderly Patients with COVID-19 hospitalised in Indonesia’s National General Hospital. *Acta Med. Indones.* 2020; **52**(3): 199–205

 5.Write the number of ethical approval: 455/X/2021/KEPK

 6. The sudden changes situation during the covid 19 had an impact on difficulty adapting and this situation had impact on mental health of elderly.

 Reference for mental health disorder categorized are DSM V and ICD XI

 7. in this study, inclusion criteria were the population og people over 60 years old and diagnosed for the first time exposure by covid 19.

 Result:

 1. Comorbidity in this study was hypertension, diabetes mellitus, and cardiovascular disease (coronary arterial disease or cerebrovascular disease). We dont study comordities that lead to mental disorders.

 2. Table 5 and 6

 Discussion:

 1. COVID-19 infection predisposes to mental disorders induced by cytokine bodies and hyperinflammatory states. Therefore, it can cause disruption of the blood-brain barrier and ultimately inflammation of the nervous system. In the elderly, there is a susceptibility to inflammation. Hyper-inflammatory conditions affect the severity of COVID-19; the severity of COVID-19 will increase the risk of post-infection mental disorders. In addition, comorbidity in the elderly is often multi-comorbid. The number of comorbidities increases the risk of mental disorders. Table 3 shows that there is a relationship between age, the degree of disease severity, and comorbidities that increase the risk of mental disorders in the elderly after COVID-19 infection. The degree of severe illness leads to a 4.5 times higher risk of mental disorders and multi-comorbidities lead to a 2.3 times higher risk of experiencing mental disorders.

 Interferon-gamma related to hyperinflammatory condition increase during covid-19 infection can affect brain function. it is well-known that chronic accumulation of cytokines causes neuronal damage

 2. Other factors like glucose level, hypertension, and other comorbidities increase the severity of the disease. Immune signatures correlated with diseases trajectory to sequalae

 3. In this study, there is no evaluation on gastrointestinal symptoms during hospitalization

 4. Decrease appetite, digestive disturbances, increased metabolism, longer duration of illness in moderate patients.
